# Connexin43 Modulates Cell Polarity and Directional Cell Migration by Regulating Microtubule Dynamics

**DOI:** 10.1371/journal.pone.0026379

**Published:** 2011-10-14

**Authors:** Richard Francis, Xin Xu, Hyunsoo Park, Chin-Jen Wei, Stephen Chang, Bishwanath Chatterjee, Cecilia Lo

**Affiliations:** 1 Genetics and Development Biology Center, National Heart Lung and Blood Institute, Bethesda, Maryland, United States of America; 2 Department of Developmental Biology, University of Pittsburgh School of Medicine, Pittsburgh, Pennsylvania, United States of America; University Hospital Hamburg-Eppendorf, Germany

## Abstract

Knockout mice deficient in the gap junction gene connexin43 exhibit developmental anomalies associated with abnormal neural crest, primordial germ cell, and proepicardial cell migration. These migration defects are due to a loss of directional cell movement, and are associated with abnormal actin stress fiber organization and a loss of polarized cell morphology. To elucidate the mechanism by which Cx43 regulates cell polarity, we used a wound closure assays with mouse embryonic fibroblasts (MEFs) to examine polarized cell morphology and directional cell movement. Studies using embryonic fibroblasts from Cx43 knockout (Cx43KO) mice showed Cx43 deficiency caused cell polarity defects as characterized by a failure of the Golgi apparatus and the microtubule organizing center to reorient with the direction of wound closure. Actin stress fibers at the wound edge also failed to appropriately align, and stabilized microtubule (Glu-tubulin) levels were markedly reduced. Forced expression of Cx43 with deletion of its tubulin-binding domain (Cx43dT) in both wildtype MEFs and neural crest cell explants recapitulated the cell migration defects seen in Cx43KO cells. However, forced expression of Cx43 with point mutation causing gap junction channel closure had no effect on cell motility. TIRF imaging revealed increased microtubule instability in Cx43KO cells, and microtubule targeting of membrane localized Cx43 was reduced with expression of Cx43dT construct in wildtype cells. Together, these findings suggest the essential role of Cx43 gap junctions in development is mediated by regulation of the tubulin cytoskeleton and cell polarity by Cx43 via a nonchannel function.

## Introduction

Gap junctions are specialized cell junctions that contain hydrophilic membrane channels that allow the passive diffusion of ions and small molecules between cells [1], [2],[3]. They are encoded by a multigene family known as the connexins. All connexins exhibit a conserved protein structure comprising of four transmembrane domains, and a cytoplasmic localized carboxy terminus with important regulatory functions [1], [2], [3]. Connexins are widely expressed, with most cells and tissues expressing multiple connexin isotypes. Analyses of knockout mice suggest different connexins may have unique functions in specific cells and tissues. For example, Cx40 have been shown to have an essential role in cardiac conduction [4], while Cx43 is critically important for cardiovascular development [5], [6], [7], [8], [9]. While the role of Cx40 in heart conduction is likely mediated by electrical coupling via the gap junction channel [4], the role of Cx43 in heart development is still not well understood.

Connexin 43 knockout (KO) mice die shortly after birth from cardiac defects associated with pulmonary outflow obstruction and coronary anomalies [5], [6], [7], [8], [9], defects that arise from the abnormal deployment of cardiac neural crest and epicardially derived cells [5], [6], [7], [8], [10], [11], [12], [13]. These are two migratory cell populations of extracardiac origin that must migrate into the embryonic heart to support normal heart development. While the cardiac neural crest cells play an essential role in the patterning of coronary arteries and outflow tract [14], the epicardially-derived cells generate the endothelial and smooth muscle cells of the coronary arteries [15].

In the Cx43 KO mouse, abnormal patterning of the coronary arteries are accompanied by outflow obstruction associated with conotruncal pouch tissue containing ectopic and disorganized deployment of endothelial and vascular smooth muscle cells [5], [6], [7], [9]. Cardiac neural crest and epicardial cells both express Cx43 and are well coupled by gap junctions [7], [8]. Using time-lapse video microscopy, we previously showed Cx43 deficiency caused significant perturbation in neural crest and epicardial cell migration [7], [10], [11], [12], [13], [16]. In addition, we showed transgenic mice overexpressing Cx43 or expressing a dominant negative Cx43-lacZ fusion protein exhibited defects in neural crest cell migration [17], [18]. Consistent with our findings, studies by others have shown a role for Cx43 in the modulation of cell motility in various tissue culture and tumor cell lines [19], [20], [21], [22], [23], [24], [25]. Together these findings support the notion that Cx43 has an important role to play in cell motile behavior. However, Cx43 has been shown to either stimulate or inhibit cell migratory behavior in different biological contexts. In keratinocytes, reducing Cx43 expression elevated wound healing with increased kerationocyte migration in both healthy [26], [27] and diabetic animal skin models [28]. Paradoxically, neuronal cell migration is impaired with similar reductions in Cx43 expression [19], [20], , while elevating Cx43 expression in cancer cells increased cell migration [30], [31]. Finally, a recent study demonstrated common Cx43 mutations associated with ODDD can result in significant reductions in cell migration and wound healing, while cell surface Cx43 expression can remain unchanged (p.D3N) or become significantly reduced (p.V216L) [32]. Cumulatively, these findings suggest an important role for Cx43 in the modulation of cell migration, one that is likely complex and is the focus of the current study.

While the importance of Cx43 in cell motility is well documented, the underlying mechanism remains unclear, and whether this requires the gap junction channel is unknown. Directional cell migration requires polarized alignment of the cytoskeleton with the microtubule organizing center (MTOC) and Golgi positioned forward facing at the cell's leading edge [13], [16], [33], [34]. We previously showed Cx43KO cells failed to realign their MTOC and Golgi with the direction of cell migration [13], [16]. This was accompanied by a loss of stabilized microtubules, which is required for establishing cell polarity. In addition, we also observed alteration in the organization of the actin cytoskeleton and focal adhesion contacts essential for directional cell migration [13], [16]. Together, these observations suggest Cx43 may modulate cell motile behavior through modulation of the cytoskeleton. Interestingly, our analysis of multiple transgenic and knockout mouse lines have found no correlation between the level of gap junctional coupling and cell motile behavior [8], [10], [11], [12], [13], [18], [35]. Similar findings have been reported in several tissue culture cell line studies [19], [36]. In light of these findings, the question that has emerged is whether Cx43 modulation of cell motility may involve a distinct non-channel function of the protein.

Cx43 interactions with the cytoskeleton have been suggested by several previous studies. The Cx43 carboxy terminus (CT) has been shown to bind ZO-1 [37], a scaffold protein that facilitates linkage of the membrane with the actin cytoskeleton [38], [39]. Cx43/ZO-1 interaction has been shown to modulate trafficking and turnover of Cx43 [40]. In Cx43KO epicardial cells, a redistribution of cell surface localized ZO-1 to the cytoplasm is observed in conjunction with disruption in organization of the epithelial sheet [16]. More intriguing is the finding that Cx43 can directly bind tubulin [37], [41]. This is mediated via amino acid residues 234–243 in the juxtamembrane region of the carboxy terminus of Cx43 [41]. Microtubules have previously been identified as playing a critical role in Cx43 trafficking to the cell membrane [42], and this trafficking has been seen to rely on the Cx43 tubulin binding domain [43], [44], [45], [46]. Previous studies showed microtubules extend preferentially to Cx43 gap-junction plaques at the cell surface, and remain at these sites longer [45]. Whether such Cx43-microtubule interactions or the Cx43 tubulin binding domain may play a role in directional cell migration has not been examined.

To investigate whether modulation of cell polarity and polarized cell migration by Cx43 may involve Cx43 regulation of microtubule dynamics, we used a wound healing assay with mouse embryonic fibroblasts (MEFs) and NIH3T3 cells to quantitatively assess cell polarity and directional cell migration. Using this wound healing assay, we also examined the requirement for the previously identified Cx43 tubulin binding domain with forced expression of a Cx43 construct with the tubulin binding domain deleted (Cx43dT). To further assess the requirement for the gap junction channel in Cx43 modulation of cell motility, we examined the expression of a Cx43 mutation (Cx43Y17S) recovered from an oculodentodigital dysplasia (ODDD) patient. This Cx43 mutant protein was previously shown to have no gap junction channel activity, but nevertheless can make gap junction plaques at the cell surface [47]. We also used TIRF imaging with tubulin-GFP to directly assess tubulin dynamics in Cx43 KO MEFs and in cells expressing Cx43dT-dsRED constructs missing the tubulin binding domain. Together these studies showed Cx43 modulates microtubule dynamics and this requires the tubulin binding domain but not cell-cell communication mediated by the Cx43 gap junction channel.

## Results

### Cx43 deficiency causes defects in directional cell migration

**Figure 1:**
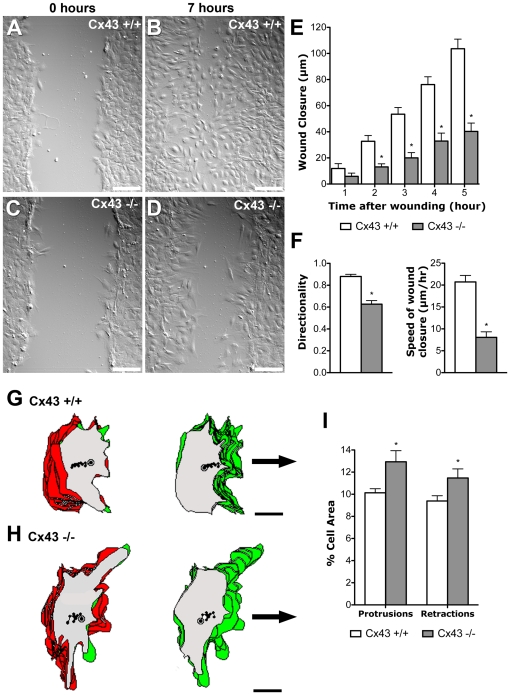
Wound closure assay shows defect in polarized cell movement in Cx43 KO MEFs. (A–F). A wound scratch was introduced in confluent monolayers of Cx43 KO (n = 7 scratches) (C) or wildtype MEFs (n = 7 scratches) (A). After 7 hrs, wildtype MEFs have migrated into the gap to close the wound (B), while there are still extensive gaps in the wound edge of KO MEFs (D). Tracking the position of the advancing wound edge revealed a significant decrease in the rate of wound closure in the KO MEFs (E). There was also a marked decrease in the directionality and speed of wound closure (F). Asterisks indicate p<0.05 when comparing wildtype vs. KO MEFs. (G–I). Tracings of individual cells at the wound edge showed a distinct polarized cell morphology associated with wildtype cells (n = 42 cells) (G), with cytoplasmic protrusions (green) concentrated at the leading edge of the cell, facing the wound edge, while retractions of cell processes (red) were situated mostly at the ipsilateral or trailing edge of the cell. In contrast, in KO MEFs (n = 42 cells) (H), cytoplasmic protrusions and retractions were observed around the entire cell circumference, with extensive overlap between regions of protrusions and retractions, thereby indicating a defect in polarized or directional cell movement. Quantitative analysis showed this was associated with an increase in both cytoplasmic protrusions and retractions (I). Data presented as mean ± SEM. Scale bars in (A–D) represent 100 µm. Scale bars in (G, H) represent 10 µm.

To assess the role of Cx43 in directional cell migration, we generated primary mouse embryonic fibroblasts (MEFs) from Cx43 wildtype and KO mouse embryos for assessment of cell motility using a well described wound healing assay [33], [48], [49]. Briefly, this entailed growing the MEFs to confluence followed by 48 hrs of serum starvation. Then a scratch is introduced across the monolayer to generate a small wound or gap, and serum is restored which stimulates cell migration across the gap to close the wound. Typically with wildtype cells, the gap is quickly filled in a few hours. However, with Cx43KO MEFs, wound closure occurred more slowly (Fig 1). Quantitation of the rate of wound closure by measuring the advancing wound edge showed wildtype MEFs migrated at an average rate of 20.7±1.5 um/hour, as compared to 8.1±1.3 um/hour for the Cx43 KO MEFs (Fig. 1E). We quantitatively assessed the directionality of cell movement at the wound edge by tracking the motion of individual cells at the migration front and measuring the total distance traveled and the net displacement achieved to calculate the directionality of cell movement (net displacement divided by total distance traveled). Cx43 KO MEFs exhibited a directionality of 0.63±0.03 vs. 0.88±0.02 for wildtype MEFs, with 1 corresponding to cell migration in a straight line across the gap. (Fig. 1F). Despite the reduced directionality of cell locomotion, the KO MEFs showed increased cell protrusive activity (Fig. 1G–I). Thus the decrease in the rate of wound closure is not due to an overall reduction in cell motility.

### Cx43 deficiency causes defects in cell polarity with loss of stabilized microtubules

**Figure 2:**
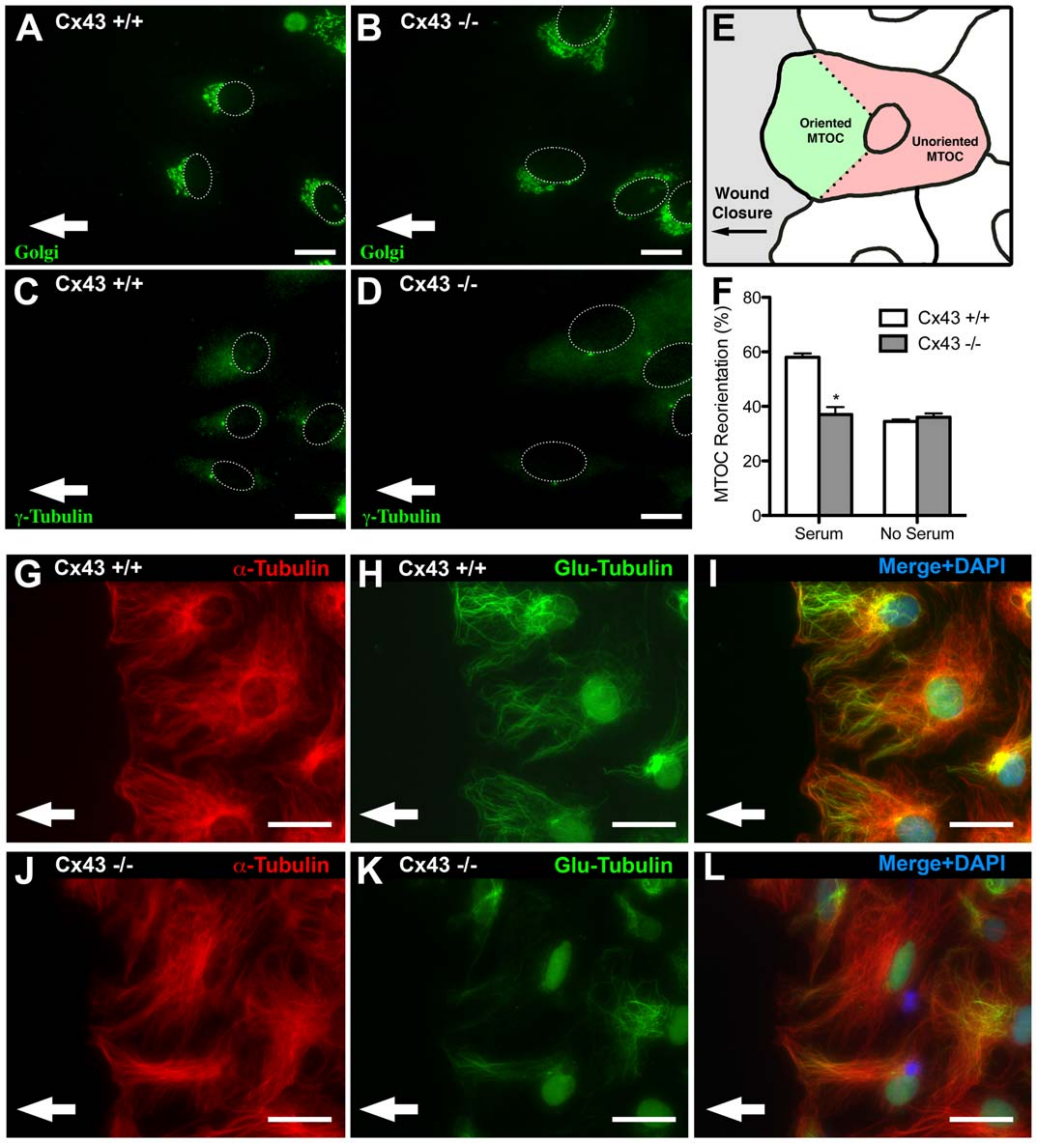
Microtubule organizing center, Golgi apparatus, and microtubules fail to reorient in Cx43α1 KO MEFs, which also display a reduction in stabilized microtubules. (A–D). The polarity of Cx43 wildtype and KO MEFs (n = 75 and 67 cells respectively) at the wound edge were examined by immunostaining with a GM130 antibody (A, B) to delineate the Golgi apparatus, and with a γ-tubulin antibody to delineate the microtubule organizing center (C, D). In wildtype cells (A,B), the Golgi and the MTOC were usually forward facing relative to the direction of wound closure (white arrow), but in KO MEFs (C,D), the position of the Golgi and MTOC appear to be randomized. (E,F). Orientation of the MTOC/Golgi was scored with each cell divided into one 120 degree sector facing the lead edge, and a second sector comprising the remainder 240 degrees (E). Cells with MTOCs located in the sector facing the lead edge were considered oriented and scored 1, cells with MTOCs positioned outside of this were considered not oriented and scored 0. Compilation of such scoring showed a significant reduction in the reorientation of the MTOC in Cx43 KO MEFs when compared to the wildtype MEFs, and this reduction was not statistically different when compared with cells with non-orientated MTOC when cultured in the absence of serum (F). These observations suggest KO MEFs have a defect in reorientation of the MTOC at the wound edge. (G–L) Immunostaining with an α-tubulin antibody showed microtubules align with the direction of wound closure in wildtype MEFs, but not in KO MEFs (G vs J). Much of the microtubules in wildtype MEFs were also immunostained by a Glu-tubulin antibody (H), while little or no Glu-tubulin staining was observed in the KO MEFs (K). Data presented as mean ± SEM. All scale bars represent 25 µm.

Cells undergoing directional cell locomotion exhibit a polarized cell morphology that is essential for productive cell migration. Typically the Golgi apparatus and microtubule-organizing center (MTOC) are situated in the forward facing direction or leading edge of the cell and the microtubule cytoskeleton is aligned with the direction of cell migration [50], [51]. We examined the polarity of wildtype and KO MEFs at the closing wound edge using GM130 antibodies to track the position of the Golgi apparatus and γ-tubulin antibodies to localize the MTOC (Fig. 2A–F). Wildtype MEFs displayed the expected polarized distribution of the Golgi/MTOC in the leading edge of the cell, aligned with the direction of wound closure (Fig. 2A,C). However, in the Cx43 KO MEFs, the Golgi and MTOC localization were randomized in orientation (Fig. 2B,D), similar to serum starved wildtype MEFs not stimulated to undergo cell migration (Fig. 2F). These results show Cx43 deficiency causes a defect in cell polarity. Immunostaining with an α-tubulin antibody showed in wildtype MEFs, microtubules were mostly aligned with the direction of wound closure (Fig. 2G), but in the Cx43 KO MEFS, microtubule orientation was randomized (Fig. 2J). Also essential for establishing cell polarity and directional cell migration is stabilization of microtubules mediated by the detyrosination of α-tubulin at the cell's leading edge [52] and positioning of Golgi at the cell's leading edge to facilitate protein and membrane synthesis required for cell motility (for review see, [51]. Stabilized microtubules can be detected with antibodies to Glu-tubulin, which detects the exposed N-terminal glutamate residue in detyrosinated tubulin [48], [49], [53]. Glu-tubulin was observed to be markedly reduced in Cx43KO vs. wildtype MEFs (Fig. 2H, K). This loss of stabilized microtubules is consistent with the defects observed in cell polarization and directional cell migration.

### Cx43 tubulin binding domain required for directional cell migration

The perturbation of the microtubule cytoskeleton in the Cx43 KO MEFs is likely central to the cell migration defects observed with Cx43 loss, and in this regard, it is interesting to consider the potential role of a previously identified Cx43 tubulin binding domain [41], [54]. To examine whether this Cx43 tubulin binding domain may be important in cell motility, we generated a Cx43 expression vector in which the tubulin binding domain was deleted (Cx43dT). To facilitate live cell tracking, we added a C-terminal in-frame fusion of either GFP or dsRED (Cx43dT-GFP, Cx43dT-DsRed). In parallel, control constructs were generated encoding wildtype full length Cx43 similarly fused in frame with either GFP or DsRed (Cx43FL-GFP, Cx43FL-DsRed).

**Figure 3:**
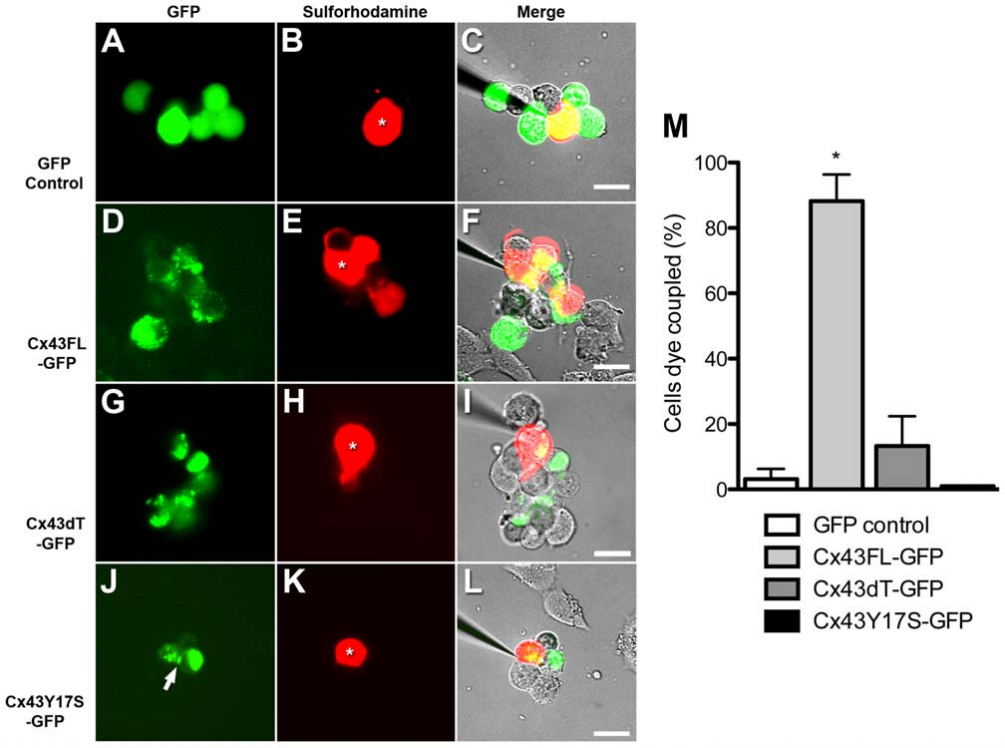
Gap junction communication competency of Cx43 constructs assayed by dye injection analysis. GFP, Cx43FL-GFP, and Cx43dT-GFP constructs were transfected into N2A cells, a cell line which does not express Cx43 and is not dye coupled. Iontophoretic injection of sulforhodamine (red) into N2A cells transfected with GFP expressing plasmid showed no dye transfer (n = 5) (A-C). In contrast, N2A cells transfected with the Cx43FL-GFP plasmid (n = 17) showed abundant dye transfer between the injected cell (denoted by asterisk) and surrounding cells (D–F). However, N2A cells transfected with either the Cx43dT-GFP plasmid (n = 15) (G–I) or the Cx43Y17S-GFP (n = 10) (J–L) plasmid did not display any dye transfer from the site of injection (see asterisks in H, K). Quantitation of the extent of dye transfer showed only N2A cells transfected with the Cx43FL-GFP plasmid acquired significant dye-coupling (n = 17) (M). Asterisks denote injected cells. Data presented as mean ± SEM. Scale bars represent 20 µm

To determine whether these Cx43 fusion protein constructs could function normally, we transfected these expression vectors into gap junction deficient N2A cells and dye coupling was assessed following iontophoretic injection of sulforhodamine 101, a low molecular weight fluorescent tracer that can pass through gap junction channels. As expected, dye injection into N2A cells expressing a GFP control plasmid showed no dye transfer from the site of injection (Fig. 3A–C, M). In contrast, N2A cells expressing full-length Cx43-GFP showed significant sulforhodamine transfer from the site of injection into surrounding cells (Fig. 3D–F, M). This same result was observed with expression of a Cx43FL-dsRED construct (data not shown). In contrast, dye injection into N2A cells expressing the Cx43dT-GFP construct did not show dye transfer (Fig. 3G–I, M), but this is presumably due to inability of the Cx43dT-GFP fusion protein to traffic to the cell surface. Previous studies have shown Cx43-microtubule interactions are required for Cx43 cell surface trafficking [43]. Consistent with this, cell surface trafficking of Cx43dT-GFP was rescued when this construct was transfected into NIH3T3 cells expressing endogenous Cx43; presumably this is mediated by formation of hetero-oligomers between endogenous Cx43 and the Cx43dT-GFP fusion protein (Figure 5G). Together these findings suggest the Cx43-GFP and Cx43dT-GFP fusion proteins can both generate gap junction plaques at the cell surface.

**Figure 4:**
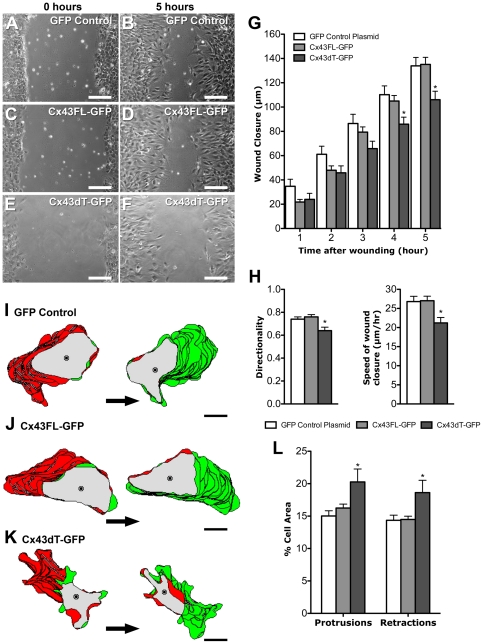
NIH3T3 cells expressing Cx43dT-GFP show defects in wound closure and polarized cell movement. (A–H) NIH3T3 cells transfected with plasmids expressing GFP, Cx43FL-GFP or Cx43dT-GFP were examined for wound closure 5 hrs after a wound scratch was made (n = 10, 10, and 8 scratches respectively). The rate of wound closure in the Cx43dT-GFP transfected cells (E, F) was reduced when compared with the GFP (A,B) or Cx43FL-GFP transfected cells (C,D). Quantitative assessment showed a significant decrease in wound closure rate in the Cx43dT-GFP expressing NIH3T3 cells at 4 and 5 hrs after wounding (G) (p<0.05). This was associated with a significant decrease in both the directionality and speed of wound closure (H). (I–L). Motion analysis with the tracing of individual cells at the wound edge showed a distinct polarized cell morphology in cells expressing GFP (n = 20 cells) (I) or Cx43FL-GFP (n = 20 cells) (J), with cytoplasmic protrusions (green) seen at the cell's leading edge facing the wound, and retracting cell processes (red) in the cell's trailing edge at the ipsilateral side. In contrast, in cells expressing the Cx43dT-GFP construct (n = 22 cells) (K), cell protrusions and retractions were not as distinctly polarized. This defect in cell polarity in cells expressing the Cx43dT-GFP construct was associated with an overall increase in cell protrusive activity (L). Data presented as mean ± SEM. Scale bars in (A–F) represent 100 µm. Scale bars in (G–I) represent 10 µm.

**Figure 5:**
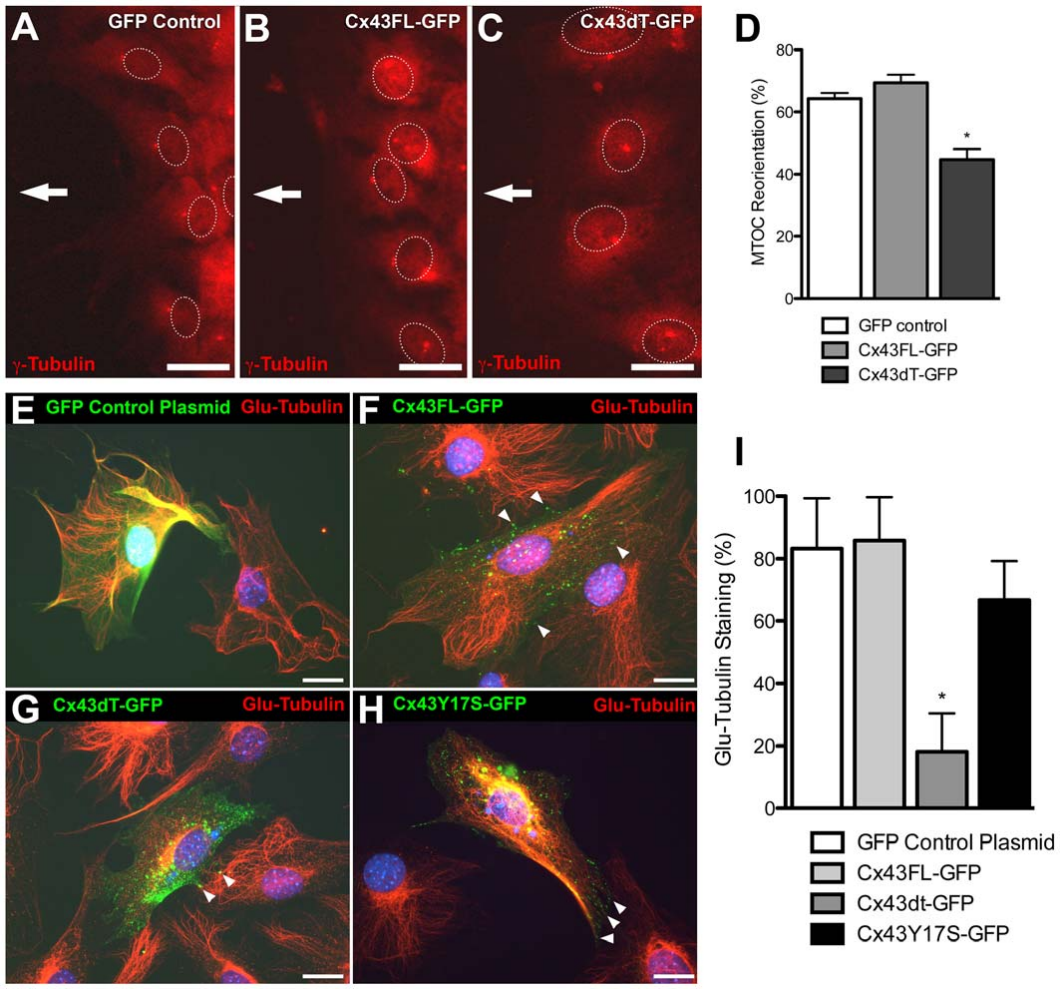
Cells expressing Cx43dT construct fail to reorient the microtubule organizing center and display loss of stabilized microtubules. (A–D) The MTOC in NIH3T3 cells stably transfected with plasmids expressing GFP (A), Cx43FL-GFP (B), or Cx43dT-GFP was visualized using γ-tubulin immunostaining (n = 481, 390, and 396 cells respectively), with the direction of wound closure indicated by the white arrow. In contrast to cells expressing GFP (A) or Cx43FL-GFP (B) plasmids, the MTOC in cells expressing Cx43dT-GFP (C) were often not aligned with the direction of wound closure. This is indicated by the quantitation showed in (D) (asterisk indicate p<0.05). (E–I) Glu-tubulin antibody staining showed comparable levels of stabilized microtubules in cells expressing GFP (E), Cx43FL-GFP (F), and Cx43Y17S-GFP (H), while cells expressing Cx43dT-GFP showed little or no Glu-tubulin staining (G). Quantitative assessment of the immunofluorescence staining shown in (I) confirmed a significant decrease only in cells expressing Cx43dT-GFP (n = 49 cells) compared with cells expressing GFP, Cx43FL-GFP, or Cx43Y17S-GFP (n = 55, 47, and 15 cells respectively). Note fluorescent gap junctional plaques are seen in cells expressing Cx43FL-GFP, Cx43Y17S-GFP, and Cx43dT-GFP (see white arrowheads), with noticeably fewer such plaques seen in cells expressing the Cx43dT-GFP. Data presented as mean ± SEM. Scale bars represent 25 µm.

To determine whether the Cx43dT protein missing the tubulin binding domain may affect cell motile behavior, we transfected NIH3T3 cells with the Cx43dT-GFP, Cx43FL-GFP, or Cx43-GFP constructs and examined cell motile behavior using the same wound closure assay. NIH3T3 cells expressing the Cx43dT-GFP protein displayed a reduced rate of wound closure (Fig. 4E–G) when compared to GFP or Cx43FL-GFP expressing NIH3T3 cells (Fig. 4A–D, G). Time lapse imaging and motion analysis of cells migrating at the wound edge showed a significant decrease in both the directionality and speed of cell locomotion in the Cx43dT-GFP expressing cells when compared with cells expressing either GFP or Cx43FL-GFP (Fig. 4H–K). While the Cx43dT-GFP expressing cells showed reduced cell locomotion, this was accompanied by increased cytoplasmic protrusions and retractions (Fig. 4K, L). These results suggest the tubulin-binding domain of Cx43 is required for directional cell locomotion, but not cell motility. Consistent with the reduction in directional cell migration, we also observed cells expressing Cx43dT-GFP were compromised in their ability to reorient their MTOC when compared with cells expressing GFP or Cx43FL-GFP constructs (Fig. 5A–D). A reduction in Glu-tubulin staining was observed in NIH3T3 cells expressing Cx43dT-GFP as compared to cells expressing GFP or Cx43FL-GFP (Fig. 5E–G, I), indicating a reduction in stabilized microtubules. Together, these results show the Cx43 tubulin-binding domain is required for directional cell migration and this entails a role in establishing polarized cell morphology.

### Cx43 maintenance of stabilized microtubules does not require gap junction channel

To examine the role of the gap junction channel in the modulation of cell polarity and directional cell migration we generated a GFP C-terminal tagged Cx43 construct with a point mutation (Cx43Y17S-GFP) that eliminates gap junction channel function without perturbing protein trafficking or assembly of gap junction plaques at the cell surface [47]. This mutation was recovered from a patient with oculodentodigital dysplasia (ODDD) [47]. We transfected the Cx43Y17S-GFP construct into gap junction communication deficient N2A cells to evaluate its ability to mediate gap junctional coupling [55]. When N2A cells expressing the mutant Cx43Y17S-GFP protein were injected with dye, there was no dye transfer (Fig. 3J–M). This is despite the localization of fusion protein at cell-cell interface consistent with gap junction plaque formation (arrow in Fig. 3J). These results confirm the previously published finding that the Cx43Y17S mutation does not affect gap junction formation, but rather gap junction communication competency [47].

To assess whether the mutant Cx43Y17S-GFP protein may alter microtubule dynamics, we examined the abundance of stabilized microtubules with Glu-tubulin immunostaining in NIH3T3 cells transfected with the Cx43Y17S-GFP construct. Cells expressing the Cx43Y17S-GFP construct showed no change in the level of Glu-tubulin expression (Fig. 5H, I) as compared to NIH3T3 cells expressing either GFP (Fig. 5E, I) or wildtype Cx43-GFP (Fig. 5F, I). This contrasts with cells expressing Cx43dT-GFP, which showed a marked reduction in Glu-tubulin (Fig. 5G, I). Together, these results suggest gap junction communication is not required for Cx43 modulation of microtubule dynamics.

### Cell migration defects in neural crest cells expressing Cx43dT-GFP

**Figure 6:**
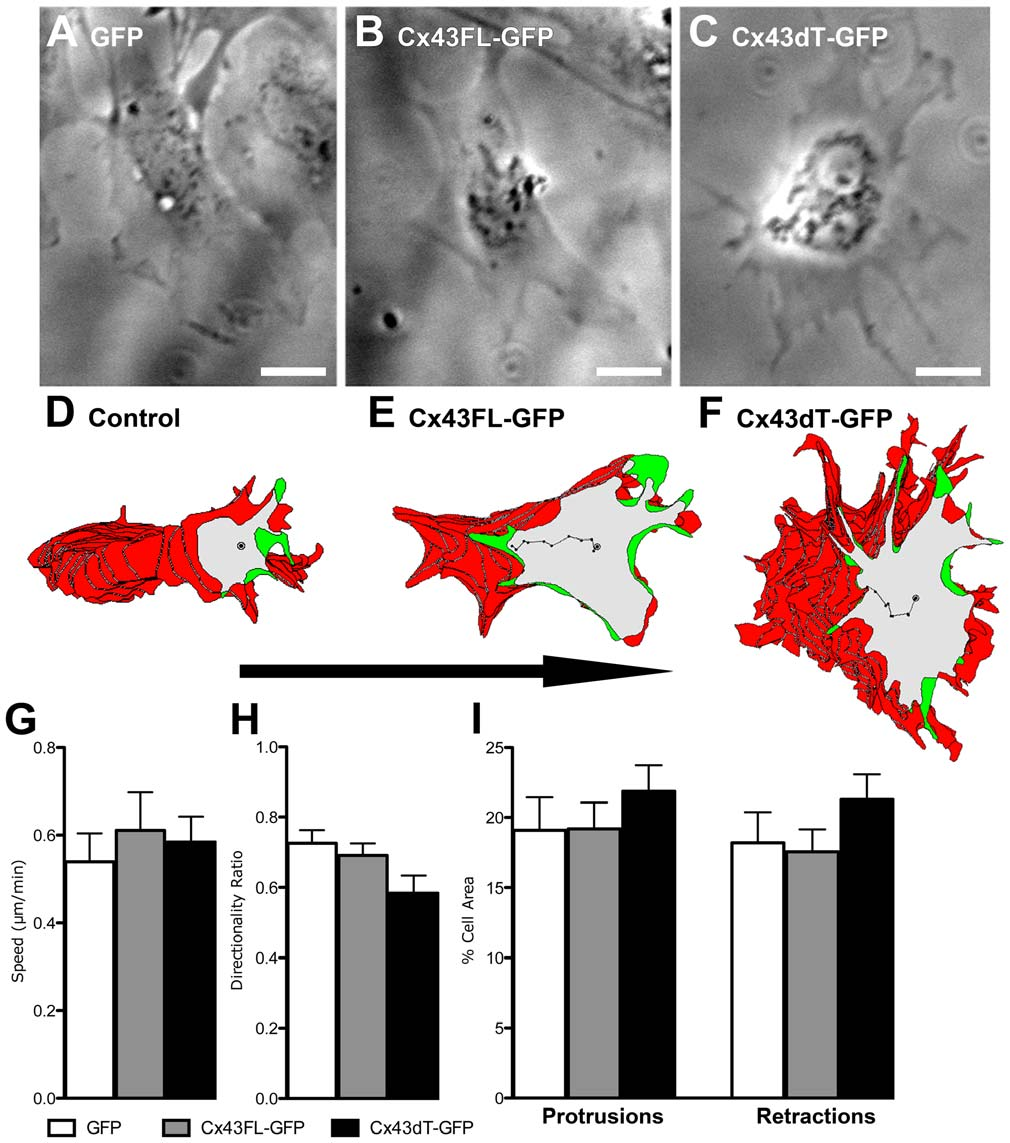
Neural crest cells expressing Cx43dT-GFP also show defects in polarized cell migration. Neural crest cell explants from E8.5 wildtype mouse embryos were transfected with either Cx43FL-GFP or Cx43dT-GFP plasmid constructs and neural crest cell migration behavior was examined 24 hours after transfection. Motion analysis with the tracing of individual cells at the explants edge showed a distinct polarized cell morphology in GFP construct-transfected control cells (n = 11 cells) (A, D) or Cx43FL-GFP transfected cells (n = 12 cells) (B, E), with cytoplasmic protrusions (green) seen at the cell's leading edge, and retracting cell processes (red) in the cell's trailing edge at the ipsilateral side. In contrast, in cells expressing the Cx43dT-GFP construct (n = 21 cells) (C, F), cell protrusions and retractions were not as distinctly polarized. This defect in cell polarity in neural crest cells expressing the Cx43dT-GFP construct was associated with an overall increase in cell protrusive activity (I), a significant decrease in cell directionality (H) (i.e. a more randomized migration pathway), and a not significantly altered migration speed (G). Scale bars = 20 µm. Data presented as mean ± SEM.

To determine if Cx43 is also required for the modulation of cell migration in neural crest cells, we explanted neural crest cells from the E8.5 postotic hindbrain neural tube, the region where cardiac crest cells emerge (Fig. 6). Explant cultures were transfected with the Cx43FL-GFP or Cx43dT-GFP plasmid and 24 hrs later neural crest cell migration was examined. In GFP construct transfected controls, motion analysis of individual neural crest cells emerging from the explants showed a distinct polarized cell morphology (Fig. 6A, D). This was also observed in explants expressing the Cx43FL-GFP construct (Fig. 6B, E). In contrast, neural crest cells expressing the Cx43dT-GFP construct (Fig. 6C, F), though exhibiting more cell protrusive activity (Fig. 6I), had cell protrusions/retractions that were not aligned with the direction of cell migration (Fig. 6F). Consistent with this, there was a trend for decreased directionality of cell migration in neural crest cells expressing Cx43dT-GFP (Fig. 6H) when compared to the GFP transfected controls or explants transfected with the Cx43FL-GFP constructs. This further highlights the importance of Cx43 expression level on the dynamic regulation of cell migration. In contrast to the changes in directional cell movement, the speed of cell locomotion was not significantly altered by expression of either the Cx43FL-GFP or Cx43dT-GFP constructs (Fig. 6G). Overall, these observations suggest the previously identified Cx43 tubulin binding domain is likely to play an important role in neural crest cell migration.

### Increased microtubule instability in Cx43 deficient cells

**Figure 7:**
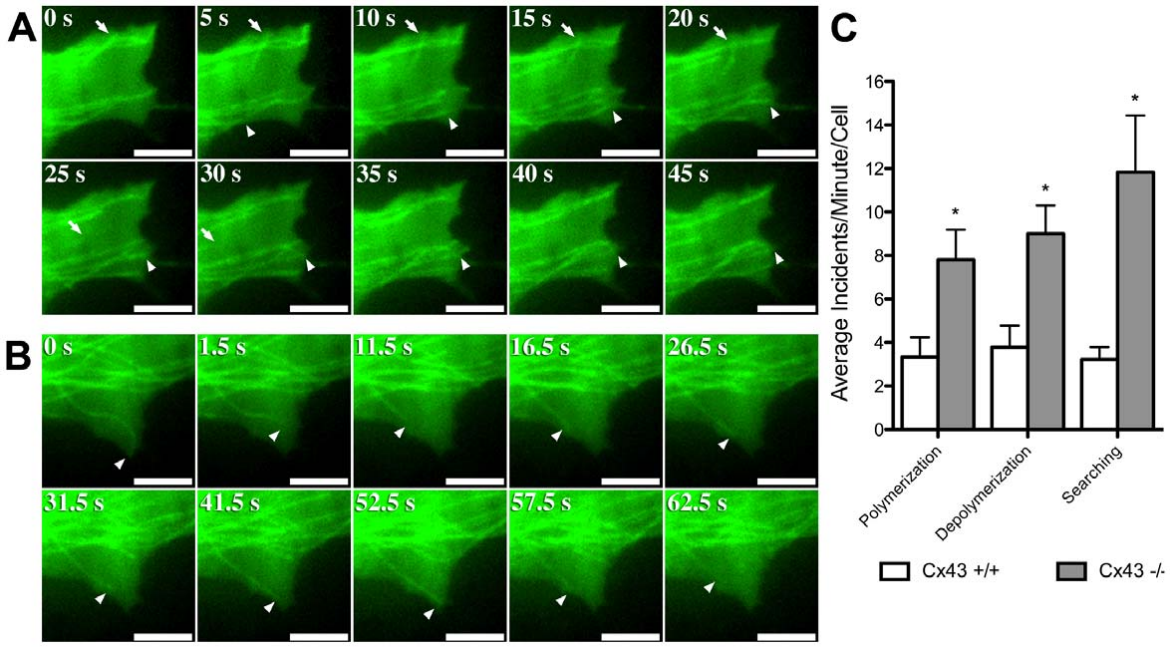
TIRF imaging show increased microtubule instability in Cx43 KO MEFs. Time lapse TIRF imaging of Cx43 KO (A) and wildtype MEFs (B) transfected with a tubulin-GFP plasmid construct (see Movie S1 and Movie S2) showed increased microtubule polymerization (arrowhead in A), depolymerization (arrow in A), and searching events (multiple polymerization/depolymerization; arrowhead in B) in the Cx43 KO MEF. Quantification of the data obtained from the TIRF imaging is shown in (C) (Cx43 +/+ n = 13 cells, Cx43 −/− n = 22 cells). Data presented as mean ± SEM. Scale bars represent 5 µm.

To delineate the role of Cx43 in the modulation of microtubule dynamics, we used TIRF microscopy to examine microtubule membrane targeting events in Cx43 KO and wildtype MEFs. Cells were transfected with a tubulin-GFP construct to allow direct visualization of microtubules (Fig. 7). We quantitatively assessed microtubule-cell membrane events and categorized them into three classes: polymerization, depolymerization, and searching events. Polymerization refers to microtubule targeting of the cell membrane (arrowhead in Fig. 7A, see Movie S1), deploymerization refers to microtubule retraction from the cell membrane (arrow in Fig. 7A), and searching refers to multiple polymerization/depolymerization events associated with a single microtubule (arrowhead in Fig. 7B, see Movie S2). Quantitation of these dynamic microtubule-cell membrane events in randomly selected cell membrane areas revealed significant increases in microtubule polymerization, depolymerization, and searching events in Cx43 KO MEFs as compared to wildtype MEFs (Fig. 7C). These results suggest an increase in microtubule instability in the Cx43KO MEFs, consistent with the observed decrease in Glu-tubulin in these cells (Fig. 2H, K).

### Cx43 tubulin binding domain promotes microtubule membrane targeting

**Figure 8:**
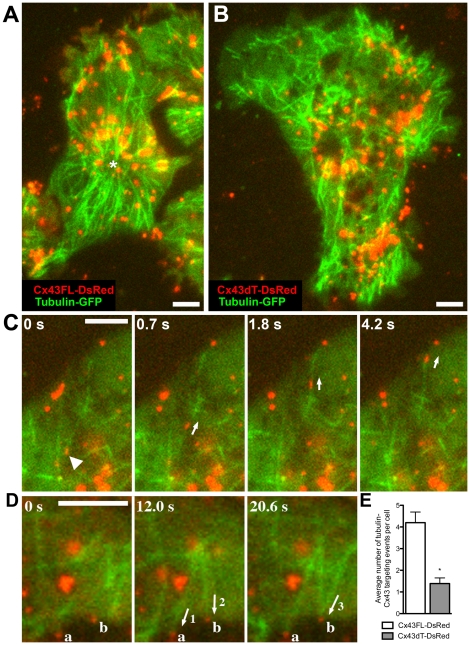
Two colour TIRF imaging show reduction in microtubule targeting to cell surface localized Cx43dT-DsRed. NIH3T3 cells transfected with GFP-tubulin and either Cx43dT-DsRed or Cx43FL-DsRed were examined by two colour TIRF imaging. In Cx43FL-DsRed expressing cells, microtubules were centrally organized around a single MTOC (asterisk in A), while in cells expressing Cx43dT-DsRed, microtubules appear disorganized in distribution (B). Time-lapse TIRF imaging shows Cx43FL-DsRed (arrowhead in C) being transported to the cell membrane along microtubules (arrows in C) (see Movie S3). Also observed is microtubule polymerization and targeting (labeled 1-3 in D) to Cx43FL-DsRed plaques in the cell membrane (labeled a, b in D) (see Movie S4). Such microtubule targeting events were significantly decreased in NIH3T3 cells expressing Cx43dT-DsRed (n = 18) when compared with those expressing Cx43FL-DsRed (n = 16) (E). Data presented as mean ± SEM. All scale bars represent 5 µm.

To examine whether modulation of tubulin dynamics by Cx43 may require the previously described Cx43 tubulin binding domain, we used two colour TIRF microscopy to directly visualize Cx43-tubulin interactions in the living cell. For these experiments, the Cx43dT-dsRED construct was transfected into NIH3T3 cells to allow cell surface trafficking of Cx43dT via hetero-oligomerization with endogenous wildtype Cx43. Control experiments with time-lapse sequences obtained by TIRF imaging of cells expressing wildtype Cx43FL-dsRED showed punctuate Cx43 red dots trafficking to the cell membrane along GFP labeled microtubules (Fig. 8C, see Movie S3). This is similar to the results previously reported [43]. In the transfected cells, Cx43FL-dsRED plaques can be seen at the cell surface and these were strong targets for capture of the polymerizing GFP labeled microtubules (Fig. 8D, see Movie S4). In cells expressing Cx43FL-DsRed, there is typically an orderly arrangement of microtubules emanating from a centrally located MTOC and the microtubules are aligned in parallel with the direction of cell migration (Fig. 8A). Similar analysis of NIH3T3 cells expressing Cx43dT-DsRed showed the Cx43dT-dsRED protein also can traffick to the cell surface along microtubules, but the incidence of microtubule capture at cell surface localized Cx43dT-dsRED was significantly decreased (Fig. 8D). In contrast to cells expressing the Cx43FL-dsRED construct, these cells displayed a disorganized pattern of microtubule distribution with no single focal origin (Fig. 8A vs B). These results suggest the tubulin-binding domain of Cx43 is required to promote microtubule targeting to the plasma membrane and for the formation of a focal MTOC.

## Discussion

Using a wound closure assay together with cell motion analysis, we showed Cx43KO MEFs have defects in directional cell migration. While the speed and directionality of cell migration were both decreased, cell protrusive activity was increased. These paradoxical results are explained by defects in cell polarity and polarized cell movement required for directional cell movement. Thus Cx43KO MEFs failed to reorient their MTOC and Golgi with the direction of cell migration and this was associated with a reduction in stabilized microtubules. MTOC/Golgi reorientation and microtubule stabilization are essential for directional cell migration (for review see, [50], [56]). We showed the regulation of the microtubule cystoeketon by Cx43 may involve the previously identified tubulin binding domain of Cx43 [37], [41].

Wildtype MEFs or NIH3T3 cells expressing the Cx43dT construct with deletion of the tubulin binding domain exhibited migration defects similar to the Cx43KO MEFs. Thus wound closure was delayed due to defects in directional cell migration, and both the speed and directionality of cell movement was reduced even as cell protrusive activity was increased. There was also a similar defect in MTOC reorientation and stabilized microtubules were reduced in abundance. The overall magnitude of these effects was less when compared to the Cx43KO MEFs. This is likely due to expression of endogenous Cx43 masking the full effects of the Cx43dT construct. We note expression of endogenous Cx43 is necessary to allow Cx43dT trafficking to the cell surface. We also showed cell-cell communication mediated by Cx43 gap junction is not essential, as cells expressing the previously identified ODDD mutation, Cx43Y17S, showed normal Glu-tubulin expression level and normal microtubule cytoskeletal organization [47].

While our findings demonstrate a reduction in Cx43 (Cx43 KO) significantly reduces cell migration in a wound closure model, they appear as odds with other wound healing assays [26], [27], [28] which show that reducing Cx43 is associated with elevated wound healing via elevated kerationocyte migration in both healthy [26], [27] and diabetic animal skin models [28]. The in vivo wound healing response is a much more complex processes involving many different cell types and signaling mechanisms, thus it's hard to state with certainty that the elevated kerationocyte migration reported in these studies was due to a Cx43 effect within the kerationocyte cells themselves or the result of external signaling factors by other cell types that may influence their migration [26], [27], [28]. It is interesting to note that other simpler in vitro experiments utilizing only a single cell type report a similar relationship between Cx43 and migration as we report here, namely reducing Cx43 results in a reduction in neuronal cell migration [19], [20], [24], [29]. Conversely, Cx43 elevation was reported to elevate cancer cell migration [22], [30], [31].

Our TIRF analysis suggests Cx43 plays an important role in regulating tubulin dynamics. Thus Cx43KO MEFs showed increased microtubule instability. These findings are consistent with previous studies showing cell surface localized Cx43 are targets for microtubule capture [41], [45]. Microtubules were reported to be three times more likely to grow to Cx43 membrane plaques where they persisted 3.5 times longer [45]. Our TIRF imaging analysis of cells expressing wildtype Cx43 and Cx43dT-dsRED showed the Cx43 tubulin binding domain is required for membrane targeting of tubulin to cell surface localized Cx43. This is consistent with previous report of the delivery of Cx43 to non-junctional area of the plasma membrane [43]. Expression of Cx43dT missing the tubulin binding domain also disrupted the normal orderly arrangement of microtubules from a centrally located MTOC, suggesting the Cx43 tubulin binding domain is essential for normal regulation of the tubulin cytoskeleton. Our observation of Cx43 trafficking to the cell surface at non cell-cell contact sites and its involvement in microtubule capture is further consistent with the notion of a non-channel function for Cx43 in the regulation of the cytoskeleton.

Overall, these findings are consistent with our previous studies showing no correlation between gap junctional communication level and changes in cell motile behavior in various transgenic and KO mouse models [13]. We previously showed neural crest cells from *Wnt1* KO mice have normal cell motility, but are not gap junction communication competent even though they had Cx43 gap junction plaques at the cell surface [11]. Based on these observations, we previously proposed that the cell surface localization of Cx43 might play a role in cell motility independent of gap junction channel activity. The results of our present study would suggest this may involve the stabilization of microtubules required for cell polarization and directional cell locomotion mediated by the tubulin binding domain of Cx43. Consistent with this, we showed expression of the Cx43dT construct missing the tubulin binding domain in neural crest cells disrupted directional cell migration, and caused cell motility defects similar to those seen in the Cx43KO neural crest cells.

Together, these findings suggest Cx43 modulation of directional cell migration involves the regulation of tubulin dynamics, while cell-cell communication mediated by the Cx43 gap junction channel may be dispensable. We propose Cx43 may have two distinct functions, one involving formation of gap junction channels to mediate direct cell-cell communication, and a separate role in modulating the cytoskeleton. The integration of these two distinct functions in one protein may be evolutionarily advantageous as it may allow efficient integration of extracellular signals with intra/intercellular cues. We note there are a plethora of studies showing interactions of Cx43 with proteins associated with the cytoskeleton, including studies showing Cx43 interactions with N-cadherin [12], [35], p120 [35], β-catenin [12], [45], vinculin [13], p150 [45], ZO-1 [57] and various actin associated proteins including drebrin [13], [19], [58], [59]. In future studies, the precise delineation of the biochemical interactions between Cx43, tubulin and other proteins that regulate the cytoskeleton will help to address the precise mechanism by which cell polarity and cell motility is regulated by Cx43.

## Methods

### Isolation of mouse embryonic fibroblasts

All experiments were conducted in accordance with an approved animal protocol of the National Heart Lung Blood Institute (Protocol Number H-0175). Mouse embryonic fibroblasts (MEF) were obtained from E13.5 day embryos generated from interbreeding heterozygous Cx43 +/− animals as previously described [60]. The protocol used for genotyping the Cx43KO mice were modified as previously reported [9] using the wildtype primer pair (IMR3: 5′-CCCCACTCTCACCTATGTCTCC-3′, IMR5: 5′-ACTTTTGCCGCCTAGCTATCCC-3′) and KO primer pair (IMR4: 5′-TAAGGGCTGGAGTTCGTGTC-3′, NEO4: 5′-ACCGCTTCCTCGTGCTTTAC-3′). MEFs were allowed to grow in standard DMEM (Invitrogen, CA) supplemented with 10% FBS (HyClone, UT) and 50 U/ug/ml penicillin-streptomycin (Invitrogen, CA).

### Wound closure assay

Confluent MEFs grown in 4-well chamber slides (BD Falcon, MA) were serum starved for 48 hr before use. The monolayer was wounded using a scratch made with a 20 ul micropipette tip for the analysis of wound closure. For time-lapse imaging, MEFs were cultured on a heated stage (37°C) in L-15 medium (+10% FBS, 50 U/ug/ml pen-strep) and imaged using a Leica inverted microscope (Leica, DMIRE2). Time-lapse images were taken every 20 minutes over 5–7 hours using 10x and/or 40x objectives using Openlab 3.1.7 software (Improvision, UK). Images were subsequently analyzed using DIAS cell tracking software (Soll Technologies, IA) to determine cell speeds, directionality, and membrane flows. Directionality was defined as the net displacement achieved divided by the total distance traveled, with a cell moving in a straight line having a directionality of one.

### Immunohistochemistry

For immunohistochemistry, cells were fixed with 4% paraformaldehyde in PBS for 10–15 min, then rinsed with PBS, permeabilized with PBST (0.15%–0.3 triton in PBS) for 10–20 minutes and blocked with 5% FBS in PBST for 1 hour. This is followed by incubation in primary antibodies in PBST (+5% FBS) overnight at 4°C, then 3 washes in PBST, and then incubation with secondary antibodies diluted in PBST for 30 minutes. After 3 washes with PBS, the cells were mounted in Vectashield with DAPI (Vector Laboratories, CA). Cells were imaged using a Leica DMIR fluorescent imaging microscope with 40x and 63x oil objectives and Orca-ER C4742-95 CCD camera (Hamamatsu, NJ). Primary antibodies used included anti-vinculin, α-tubulin, γ-tubulin, GM130 (Sigma, MO), and Glu-tubulin (Chemicon). Secondary antibodies were from Jackson Laboratories (Jackson ImmunoResearch Laboratories, PA). Orientation of the MTOC/Golgi was scored according to the methods of Gomes & Gundersen [61]. It the case of MTOC/Golgi orientation measurements of cells transfected with Cx43 plasmid constructs, only cells displaying successful plasmid transfection based on visualization of GFP fluorescence were scored.

### Plasmids

To generate GFP or DsRed-tagged Cx43 expressing vectors, cDNA encoding *Mus musculus* full-length Cx43 (amino acids 1–382) was generated by PCR and the fragment was cloned into either pcDNA3.1/CT-GFP-TOPO vector (Invitrogen, Carlsbad, CA) or pDsRed-Monomer-N1 vector (Clontech, Mountain View, CA). Cx43Y17S and Cx43-Δ234–243 (Cx43dT) mutants were generated using QuikChange II Site-Directed Mutagenesis Kit (Stratagene, La Jolla, CA) according to manufacturer's protocol. The GFP-tagged Tubulin was kindly supplied by Dr Chloe Bulinski (Columbia University, NY).

### Neural tube explant cultures

Embryos used for neural tube explant cultures were harvested at E8.5 day as described previously [13]. In brief, the hindbrain neural folds were treated with collagenase/dispase (Roche, Indianapolis, IN), and the dorsal ridge of the neuroepithelium spanning the postotic region of the hindbrain neural fold was surgically removed from the surrounding tissue and cultured overnight on plates coated with human plasma fibronectin (Life Technologies or Sigma) in Dulbecco's modified Eagle's medium (DMEM) with high glucose and 10% fetal bovine serum (FBS). The next day explants were transfected with plasmid DNA using PolyFect (Qiagen, CA).

Neural crest cell migration was subsequently recorded and analyzed 24 hours after transfection as previously described [13]. In brief, neural tube explants were cultured in phosphate buffered L-15 medium (Sigma) containing 10% FBS on a Leica DMIRE2 inverted microscope with 37°C heated stage. Timelapse images were captured using an Orca-ER camera. Quantitative motion analysis of individual cells located at the migration front of the emerging explant was carried out using Dynamic Image Analysis Software (Solltech, Oakdale, IA).

### Dye Coupling Analysis of Cx43 Constructs

Gap junction communication competency of cells expressing various Cx43 constructs was determined using Neuro-2a (N2A) cells (ATCC, VA). N2A cells where cultured on fibronectin coated 12 mm glass coverslips (Fisherbrand, Fisher Scientific, PA) and transfected with plasmid DNA using PolyFect (Qiagen, CA). 24–48 hours after transfection coverslips were placed on a heated stage of a Leica DMLFSA microscope in a dish containing prewarmed L-15 medium (+10% FBS) and dye-coupling was quantified by iontophoretic dye injection (0.5 nA current pulses at 1 Hz) of sulforhodamine 101 dye (MW 606.71, 12.5 mg/ml, Invitrogen, CA). Fluorescent images were collected every 30 seconds over 4 minutes using Cy3 filters. Dye-coupling was quantified by counting the percentage of dye containing transfected cells surrounding the injected cell.

### Total internal reflection fluorescence (TIRF) imaging

NIH3T3 (ATCC, VA) or MEF cells were plated on fibronectin coated glass bottomed culture dishes (WillCo Wells, Netherlands) and allowed to grow overnight. The next day cells were transfected with Cx43 and/or tubulin-GFP constructs using lipofectamine (Invitrogen, CA). Western blot anaylsis was used to confirm expression levels were comparable between groups (Figure S1). Cells were imaged 24–48 hours after transfection in L-15 medium (+10% FBS, pen-strep, no phenol red). Single colour time-lapse images of tubulin-GFP were collected at 2 fps on a Leica DMI 6000 B TIRF microscope with 488 nm laser, 100x NA 1.46 oil immersion objective, and a Cascade:512B EM CCD camera (Roper Scientific, AZ). Two colour time-lapse images of Cx43-DsRed and tubulin-GFP interactions were collected at ∼2.7 fps on a Leica AF 6000 LX TIRF microscope fitted with 488 nm and 561 nm lasers, 100x NA 1.46 oil immersion objective, and Hamamatsu C9100-13 ImagEM enhanced EM CCD camera (Hamamatsu, NJ). Both TIRF microscopes were fitted with incubation chambers (37°C) and movies were collected over 2 minutes using the Leica software provided.

### Statistical analysis

Data is presented as mean ± SEM as analyzed using Instat 3 (GraphPad Software, Inc.) with one-way analysis of variance (ANOVA) and Bonferroni correction. P<0.05 was considered significant.

## Supporting Information

Figure S1 Relative expression levels of Cx43 isoforms following transfection into NIH3T3 cells. Separate Cx43 and EGFP western blots were run using rabbit polyclonal antibodies. Merge image was generated by lining up fragment ladders. * highlights location of GFP tagged Cx43 (ie GFP: 27 kb+Cx43: 43 kb = ∼70 kb in total) † highlights unbound GFP protein (GFP: 27 kb).
(TIF)

Movie S1 TIRF microscopy and time lapse imaging show microtubule polymerization/depolymerization events at the cell membrane of a Cx43 KO MEF. Images from this video sequence was used to generate the panels in Figure 7A. Green fluorescence is GFP-tubulin. Frames were collected at 2 fps, playback is at 20 fps. Scale bar = 5 µm.
(MOV)

Movie S2 Time lapse imaging with single colour TIRF microscopy shows polymerization/depolymerization of a single microtubule, termed searching, at the cell membrane of a Cx43 KO MEF. Images from this video sequence was used to generate the panels in Figure 7B. Green fluorescence is GFP-tubulin. Frames were collected at 2 fps, playback is at 20 fps. Scale bar = 5 µm.
(MOV)

Movie S3 Two colour TIRF microscopy and time lapse imaging showing a Cx43FL-DsRed plaque being transported to the cell membrane along a microtubule (from bottom to top of movie) in a NIH3T3 cell. Images from this video was used to generate the panels in Figure 8C. Green fluorescence is GFP-tubulin, red fluorescence is Cx43-DsRed. Frames were collected at 2.7 fps, playback is at 10 fps. Scale bar = 5 µm.
(MOV)

Movie S4 Two colour TIRF microscopy and time lapse imaging showing microtubule polymerization and targeting of Cx43FL-DsRed plaques in the cell membrane of a NIH3T3 cell. Images from this video was used to generate the panels in Figure 8D. Green fluorescence is GFP-tubulin, red fluorescence is Cx43-DsRed. Frames were collected at 2.7 fps, playback is at 10 fps. Scale bar = 5 um.
(MOV)

## References

[1] Bukauskas FF, Verselis VK (2004). Gap junction channel gating.. Biochim Biophys Acta.

[2] Cruciani V, Mikalsen SO (2006). The vertebrate connexin family.. Cell Mol Life Sci.

[3] Wei CJ, Xu X, Lo CW (2004). Connexins and cell signaling in development and disease.. Annu Rev Cell Dev Biol.

[4] Chaldoupi SM, Loh P, Hauer RN, de Bakker JM, van Rijen HV (2009). The role of connexin40 in atrial fibrillation.. Cardiovasc Res.

[5] Clauss SB, Walker DL, Kirby ML, Schimel D, Lo CW (2006). Patterning of coronary arteries in wildtype and connexin43 knockout mice.. Dev Dyn.

[6] Walker DL, Vacha SJ, Kirby ML, Lo CW (2005). Connexin43 deficiency causes dysregulation of coronary vasculogenesis.. Developmental Biology.

[7] Li WE, Waldo K, Linask KL, Chen T, Wessels A (2002). An essential role for connexin43 gap junctions in mouse coronary artery development.. Development.

[8] Lo CW, Cohen MF, Huang GY, Lazatin BO, Patel N (1997). Cx43 gap junction gene expression and gap junctional communication in mouse neural crest cells.. Dev Genet.

[9] Reaume AG, de Sousa PA, Kulkarni S, Langille BL, Zhu D (1995). Cardiac malformation in neonatal mice lacking connexin43.. Science.

[10] Huang GY, Cooper ES, Waldo K, Kirby ML, Gilula NB (1998). Gap junction-mediated cell-cell communication modulates mouse neural crest migration.. Journal of Cell Biology.

[11] Xu X, Li WE, Huang GY, Meyer R, Chen T (2001). N-cadherin and Cx43alpha1 gap junctions modulates mouse neural crest cell motility via distinct pathways.. Cell Commun Adhes.

[12] Xu X, Li WE, Huang GY, Meyer R, Chen T (2001). Modulation of mouse neural crest cell motility by N-cadherin and connexin 43 gap junctions.. J Cell Biol.

[13] Xu X, Francis R, Wei CJ, Linask KL, Lo CW (2006). Connexin 43-mediated modulation of polarized cell movement and the directional migration of cardiac neural crest cells.. Development.

[14] Hutson MR, Kirby ML (2003). Neural crest and cardiovascular development: a 20-year perspective.. Birth Defects Res C Embryo Today.

[15] Ishii Y, Langberg J, Rosborough K, Mikawa T (2009). Endothelial cell lineages of the heart.. Cell Tissue Res.

[16] Rhee DY, Zhao XQ, Francis RJ, Huang GY, Mably JD (2009). Connexin 43 regulates epicardial cell polarity and migration in coronary vascular development.. Development.

[17] Ewart JL, Cohen MF, Meyer RA, Huang GY, Wessels A (1997). Heart and neural tube defects in transgenic mice overexpressing the Cx43 gap junction gene.. Development.

[18] Sullivan R, Huang GY, Meyer RA, Wessels A, Linask KK (1998). Heart malformations in transgenic mice exhibiting dominant negative inhibition of gap junctional communication in neural crest cells.. Dev Biol.

[19] Elias LA, Wang DD, Kriegstein AR (2007). Gap junction adhesion is necessary for radial migration in the neocortex.. Nature.

[20] Wiencken-Barger AE, Djukic B, Casper KB, McCarthy KD (2007). A role for Connexin43 during neurodevelopment.. Glia.

[21] Liu X, Liu W, Yang L, Xia B, Li J (2007). Increased connexin 43 expression improves the migratory and proliferative ability of H9c2 cells by Wnt-3a overexpression.. Acta Biochim Biophys Sin (Shanghai).

[22] Bates DC, Sin WC, Aftab Q, Naus CC (2007). Connexin43 enhances glioma invasion by a mechanism involving the carboxy terminus.. Glia.

[23] Li Z, Zhou Z, Donahue HJ (2008). Alterations in Cx43 and OB-cadherin affect breast cancer cell metastatic potential.. Clin Exp Metastasis.

[24] Cina C, Maass K, Theis M, Willecke K, Bechberger JF (2009). Involvement of the cytoplasmic C-terminal domain of connexin43 in neuronal migration.. J Neurosci.

[25] Olk S, Turchinovich A, Grzendowski M, Stuhler K, Meyer HE (2010). Proteomic analysis of astroglial connexin43 silencing uncovers a cytoskeletal platform involved in process formation and migration.. Glia.

[26] Mori R, Power KT, Wang CM, Martin P, Becker DL (2006). Acute downregulation of connexin43 at wound sites leads to a reduced inflammatory response, enhanced keratinocyte proliferation and wound fibroblast migration.. J Cell Sci.

[27] Kretz M, Euwens C, Hombach S, Eckardt D, Teubner B (2003). Altered connexin expression and wound healing in the epidermis of connexin-deficient mice.. J Cell Sci.

[28] Wang CM, Lincoln J, Cook JE, Becker DL (2007). Abnormal connexin expression underlies delayed wound healing in diabetic skin.. Diabetes.

[29] Fushiki S, Perez Velazquez JL, Zhang L, Bechberger JF, Carlen PL (2003). Changes in neuronal migration in neocortex of connexin43 null mutant mice.. J Neuropathol Exp Neurol.

[30] Pollmann MA, Shao Q, Laird DW, Sandig M (2005). Connexin 43 mediated gap junctional communication enhances breast tumor cell diapedesis in culture.. Breast Cancer Res.

[31] Zhang W, Nwagwu C, Le DM, Yong VW, Song H (2003). Increased invasive capacity of connexin43-overexpressing malignant glioma cells.. J Neurosurg.

[32] Churko JM, Shao Q, Gong XQ, Swoboda KJ, Bai D (2011). Human dermal fibroblasts derived from oculodentodigital dysplasia patients suggest that patients may have wound-healing defects.. Hum Mutat.

[33] Kupfer A, Louvard D, Singer SJ (1982). Polarization of the Golgi apparatus and the microtubule-organizing center in cultured fibroblasts at the edge of an experimental wound.. Proc Natl Acad Sci U S A.

[34] Magdalena J, Millard TH, Machesky LM (2003). Microtubule involvement in NIH 3T3 Golgi and MTOC polarity establishment.. J Cell Sci.

[35] Wei CJ, Francis R, Xu X, Lo CW (2005). Connexin43 associated with an N-cadherin-containing multiprotein complex is required for gap junction formation in NIH3T3 cells.. J Biol Chem.

[36] McLachlan E, Shao Q, Wang HL, Langlois S, Laird DW (2006). Connexins act as tumor suppressors in three-dimensional mammary cell organoids by regulating differentiation and angiogenesis.. Cancer Res.

[37] Giepmans BN, Verlaan I, Moolenaar WH (2001). Connexin-43 interactions with ZO-1 and alpha- and beta-tubulin.. Cell Commun Adhes.

[38] Hartsock A, Nelson WJ (2008). Adherens and tight junctions: structure, function and connections to the actin cytoskeleton.. Biochimica et Biophysica Acta.

[39] Itoh M, Nagafuchi A, Moroi S, Tsukita S (1997). Involvement of ZO-1 in cadherin-based cell adhesion through its direct binding to alpha catenin and actin filaments.. Journal of Cell Biology.

[40] Gourdie RG, Ghatnekar GS, O'Quinn M, Rhett MJ, Barker RJ (2006). The unstoppable connexin43 carboxyl-terminus: new roles in gap junction organization and wound healing.. Annals of the New York Academy of Sciences.

[41] Giepmans BN, Verlaan I, Hengeveld T, Janssen H, Calafat J (2001). Gap junction protein connexin-43 interacts directly with microtubules.. Curr Biol.

[42] Thomas T, Jordan K, Simek J, Shao Q, Jedeszko C (2005). Mechanisms of Cx43 and Cx26 transport to the plasma membrane and gap junction regeneration.. J Cell Sci.

[43] Lauf U, Giepmans BN, Lopez P, Braconnot S, Chen SC (2002). Dynamic trafficking and delivery of connexons to the plasma membrane and accretion to gap junctions in living cells.. Proc Natl Acad Sci U S A.

[44] Giessmann D, Theiss C, Breipohl W, Meller K (2005). Decreased gap junctional communication in neurobiotin microinjected lens epithelial cells after taxol treatment.. Anat Embryol (Berl).

[45] Shaw RM, Fay AJ, Puthenveedu MA, von Zastrow M, Jan YN (2007). Microtubule plus-end-tracking proteins target gap junctions directly from the cell interior to adherens junctions.. Cell.

[46] Guo Y, Martinez-Williams C, Rannels DE (2003). Gap junction-microtubule associations in rat alveolar epithelial cells.. Am J Physiol Lung Cell Mol Physiol.

[47] Shibayama J, Paznekas W, Seki A, Taffet S, Jabs EW (2005). Functional characterization of connexin43 mutations found in patients with oculodentodigital dysplasia.. Circ Res.

[48] Gundersen GG, Bulinski JC (1988). Selective stabilization of microtubules oriented toward the direction of cell migration.. Proc Natl Acad Sci U S A.

[49] Gundersen GG, Kim I, Chapin CJ (1994). Induction of stable microtubules in 3T3 fibroblasts by TGF-beta and serum.. J Cell Sci.

[50] Gundersen GG (2002). Evolutionary conservation of microtubule-capture mechanisms.. Nat Rev Mol Cell Biol.

[51] Mellor H (2004). Cell motility: Golgi signalling shapes up to ship out.. Curr Biol.

[52] Webster DR, Gundersen GG, Bulinski JC, Borisy GG (1987). Differential turnover of tyrosinated and detyrosinated microtubules.. Proc Natl Acad Sci U S A.

[53] Westermann S, Weber K (2003). Post-translational modifications regulate microtubule function.. Nat Rev Mol Cell Biol.

[54] Dai P, Nakagami T, Tanaka H, Hitomi T, Takamatsu T (2007). Cx43 mediates TGF-beta signaling through competitive Smads binding to microtubules.. Mol Biol Cell.

[55] Jordan K, Solan JL, Dominguez M, Sia M, Hand A (1999). Trafficking, assembly, and function of a connexin43-green fluorescent protein chimera in live mammalian cells.. Mol Biol Cell.

[56] Vinogradova T, Miller PM, Kaverina I (2009). Microtubule network asymmetry in motile cells: role of Golgi-derived array.. Cell Cycle.

[57] Taliana L, Benezra M, Greenberg RS, Masur SK, Bernstein AM (2005). ZO-1: lamellipodial localization in a corneal fibroblast wound model.. Invest Ophthalmol Vis Sci.

[58] Majoul I, Shirao T, Sekino Y, Duden R (2007). Many faces of drebrin: from building dendritic spines and stabilizing gap junctions to shaping neurite-like cell processes.. Histochemistry & Cell Biology.

[59] Squecco R, Sassoli C, Nuti F, Martinesi M, Chellini F (2006). Sphingosine 1-phosphate induces myoblast differentiation through Cx43 protein expression: a role for a gap junction-dependent and -independent function.. Mol Biol Cell.

[60] Lengner CJ, Lepper C, van Wijnen AJ, Stein JL, Stein GS (2004). Primary mouse embryonic fibroblasts: a model of mesenchymal cartilage formation.. J Cell Physiol.

[61] Gomes ER, Gundersen GG (2006). Real-time centrosome reorientation during fibroblast migration.. Methods Enzymol.

