# Supplementary figures and images for: Connexin43 Modulates Cell Polarity and Directional Cell Migration by Regulating Microtubule Dynamics
Source: PLoS One. 2011 Oct 14;6(10):e26379. doi: 10.1371/journal.pone.0026379 (PMC3194834; doi:10.1371/journal.pone.0026379)

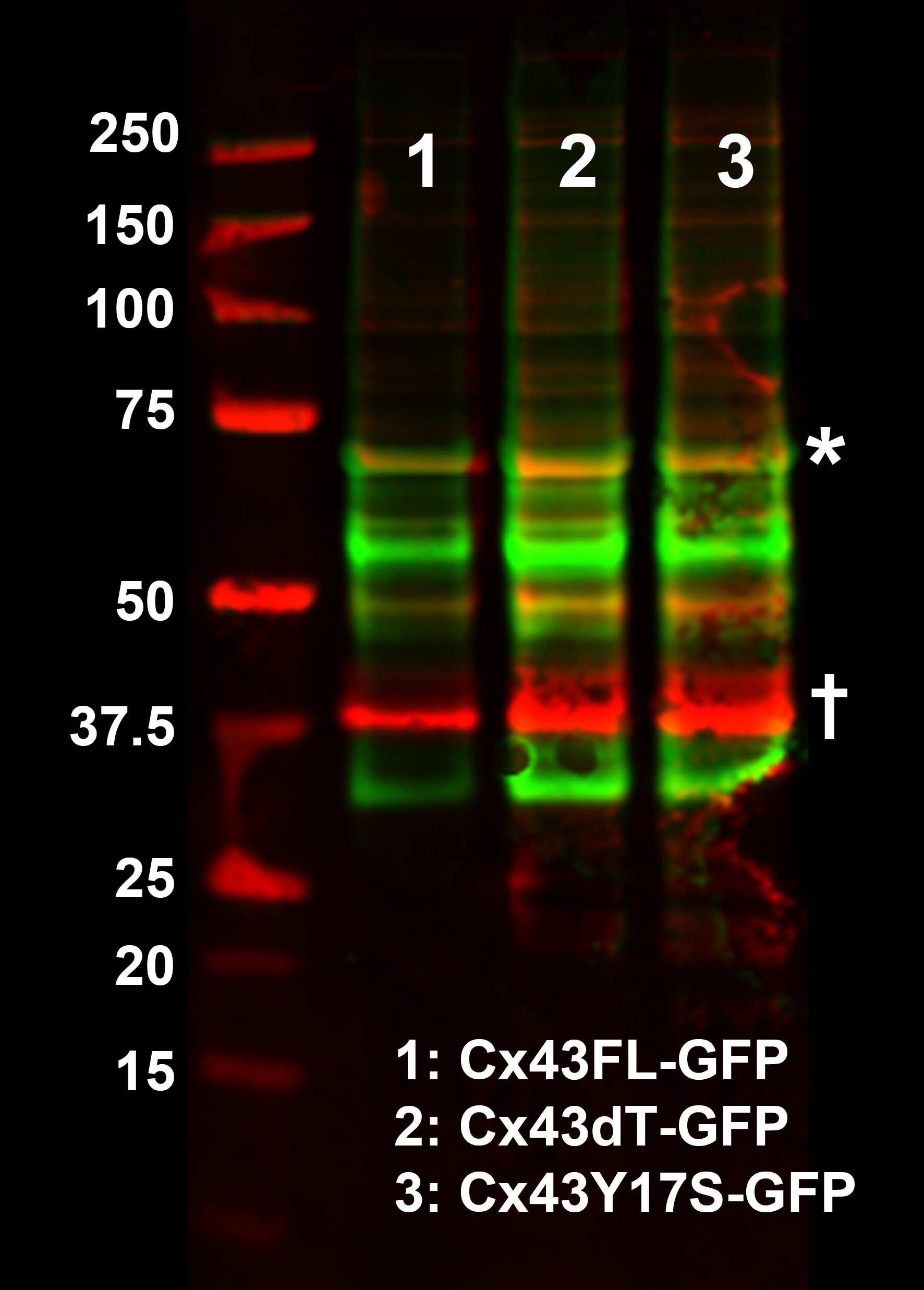

Supplement: Figure S1 — Relative expression levels of Cx43 isoforms following transfection into NIH3T3 cells. Separate Cx43 and EGFP western blots were run using rabbit polyclonal antibodies. Merge image was generated by lining up fragment ladders. * highlights location of GFP tagged Cx43 (ie GFP: 27 kb+Cx43: 43 kb = ∼70 kb in total) † highlights unbound GFP protein (GFP: 27 kb). (TIF) [file pone.0026379.s001.tif]
